# Deep Learning–based Automated Coronary Plaque Quantification: First Demonstration With Ultra-high Resolution Photon-counting Detector CT at Different Temporal Resolutions

**DOI:** 10.1097/RLI.0000000000001233

**Published:** 2025-08-22

**Authors:** Konstantin Klambauer, Silvan Daniel Burger, Tristan Thorben Demmert, Victor Mergen, Lukas Jakob Moser, Mehmet Akif Gulsun, Max Schöbinger, Chris Schwemmer, Michael Wels, Thomas Allmendinger, Matthias Eberhard, Hatem Alkadhi, Bernhard Schmidt

**Affiliations:** Diagnostic and Interventional Radiology, University Hospital Zurich, University of Zurich, Zurich, Switzerland (K.K., S.D.B., T.T.D., V.M., L.J.M., M.E., H.A.); Computed Tomography, Siemens Healthineers AG, Forchheim, Germany (M.S., C.S., M.W., T.A., B.S.); Siemens Healthineers, Princeton, NJ (M.A.G.); University Hospital Erlangen, Erlangen, Germany (B.S.)

**Keywords:** photon-counting detector CT, ultra-high resolution, coronary CT angiography, temporal resolution, deep learning, coronary plaque quantification

## Abstract

**Objectives:**

The aim of this study was to evaluate the feasibility and reproducibility of a novel deep learning (DL)-based coronary plaque quantification tool with automatic case preparation in patients undergoing ultra-high resolution (UHR) photon-counting detector CT coronary angiography (CCTA), and to assess the influence of temporal resolution on plaque quantification.

**Materials and Methods:**

In this retrospective single-center study, 45 patients undergoing clinically indicated UHR CCTA were included. In each scan, 2 image data sets were reconstructed: one in the dual-source mode with 66 ms temporal resolution and one simulating a single-source mode with 125 ms temporal resolution. A novel, DL-based algorithm for fully automated coronary segmentation and intensity-based plaque quantification was applied to both data sets in each patient. Plaque volume quantification was performed at the vessel-level for the entire left anterior descending artery (LAD), left circumflex artery (CX), and right coronary artery (RCA), as well as at the lesion-level for the largest coronary plaque in each vessel. Diameter stenosis grade was quantified for the coronary lesion with the greatest longitudinal extent in each vessel. To assess reproducibility, the algorithm was rerun 3 times in 10 randomly selected patients, and all outputs were visually reviewed and confirmed by an expert reader. Paired Wilcoxon signed-rank tests with Benjamini-Hochberg correction were used for statistical comparisons.

**Results:**

One hundred nineteen out of 135 (88.1%) coronary arteries showed atherosclerotic plaques and were included in the analysis. In the reproducibility analysis, repeated runs of the algorithm yielded identical results across all plaque and lumen measurements (*P* > 0.999). All outputs were confirmed to be anatomically correct, visually consistent, and did not require manual correction. At the vessel level, total plaque volumes were higher in the 125 ms reconstructions compared with the 66 ms reconstructions in 28 of 45 patients (62%), with both calcified and noncalcified plaque volumes being higher in 32 (71%) and 28 (62%) patients, respectively. Total plaque volumes in the LAD, CX, and RCA were significantly higher in the 125 ms reconstructions (681.3 vs. 647.8  mm^3^, *P* < 0.05). At the lesion level, total plaque volumes were higher in the 125 ms reconstructions in 44 of 45 patients (98%; 447.3 vs. 414.9  mm^3^, *P* < 0.001), with both calcified and noncalcified plaque volumes being higher in 42 of 45 patients (93%). The median diameter stenosis grades for all vessels were significantly higher in the 125 ms reconstructions (35.4% vs. 28.1%, *P* < 0.01).

**Conclusions:**

This study evaluated a novel DL-based tool with automatic case preparation for quantitative coronary plaque in UHR CCTA data sets. The algorithm was technically robust and reproducible, delivering anatomically consistent outputs not requiring manual correction. Reconstructions with lower temporal resolution (125 ms) systematically overestimated plaque burden compared with higher temporal resolution (66 ms), underscoring that protocol standardization is essential for reliable DL-based plaque quantification.

Coronary computed tomography angiography (CCTA) has become a cornerstone in the noninvasive assessment of coronary artery disease (CAD), enabling both anatomic evaluation of coronary stenosis and quantification of atherosclerotic plaque burden.^1^ Artificial intelligence, particularly deep learning (DL)-based algorithms, has been explored in research settings to improve standardization and reproducibility in both quantitative and qualitative CCTA analysis.^2^ DL-based tools for coronary plaque and stenosis quantification have shown robust performance in detecting obstructive CAD,^3^ classifying plaque components,^4^ and predicting adverse cardiovascular outcomes,^1^ with accuracy comparable to expert readers and invasive reference standards such as intravascular ultrasound.^5,6^

Photon-counting detector CT (PCD-CT) represents the most recent technological advancement in CCTA by providing ultra-high resolution (UHR) imaging with 0.2 mm voxel sizes.^7,8^ The dual-source configuration of the PCD-CT system used in this study enables image acquisition at a temporal resolution of 66 ms, which is particularly beneficial for UHR coronary plaque imaging.^9,10^

Recent studies have shown that DL-based algorithms for CCTA analysis can be sensitive to technical imaging parameters such as reconstruction kernel and denoising strength—factors that may influence output consistency, even in research tools with previously demonstrated diagnostic performance.^11^ Furthermore, many—if not most—software tools were developed and trained with energy-integrating detector (EID)-CT data and often rely on manual interaction or off-site processing.^4,5,12^

The aim of this study was to evaluate the feasibility and reproducibility of a novel DL-based coronary plaque quantification tool with automatic case preparation in patients undergoing UHR CCTA, and to assess the influence of temporal resolution on plaque quantification.

## MATERIALS AND METHODS

### Patient Population

This study complied with the Declaration of Helsinki and received local ethics committee board approval.

This retrospective study was based on a prospective registry of patients undergoing UHR CCTA for transcatheter aortic valve replacement planning. All patients had provided written informed consent for the use of their imaging data. Consecutive patients from this registry between February 2022 and July 2024 were screened for inclusion. Exclusion criteria were (i) the absence of CAD, (ii) foregoing coronary revascularization, including bypass grafting or coronary stenting, and (iii) insufficient image quality.

### CT Data Acquisition

CCTA was conducted using a first-generation dual-source PCD-CT system (NAEOTOM Alpha, Siemens Healthineers AG). UHR CCTA was performed using either prospective ECG-triggered sequential acquisition (15/45 patients, 33.3%) or retrospective ECG-gated spiral acquisition (30/45 patients, 66.6%). The choice of acquisition mode was determined by 4 cardiovascular radiologists [(K.K., V.M., M.E., H.A.), with 5, 7, 15, and 20 years of experience in cardiovascular CT, respectively] according to heart rate and rhythm: sequential acquisition was selected in patients with stable sinus rhythm and a heart rate below 70 bpm, whereas spiral acquisition was chosen for patients with irregular rhythm or heart rates of 70 bpm or higher. The tube voltage was 120 kVp, the image quality level was set at 64, and the detector collimation was 120 × 0.2 mm. The gantry rotation time was 0.25 seconds. Automatic tube current modulation (CARE Dose4D; Siemens) was used. The median volume CT dose index (CTDI_vol_) was 36.5 mGy (interquartile range: 31.0, 43.5).

Patients received 50 to 80 mL of contrast medium (iopromide, Ultravist 370 mg iodine/mL; Bayer AG) and a 20 mL saline chaser injected via an 18-gauge catheter at a weight-based flow rate of 3.3 to 4.4 mL/s. Scan triggering was performed with bolus tracking in the ascending aorta and a 140 HU threshold at 90 kV. The ECG pulsing window was fixed at 30% to 80% of the R-R interval for both sequential acquisition and spiral acquisition protocols. Sublingual nitroglycerin (2.5 mg isosorbide dinitrate) was administered before the scan unless contraindicated. No beta-blockers were used.

### CT Image Reconstruction

Images were reconstructed using dedicated research software (ReconCT Prototype, Version 16.0.1.2, Siemens Healthineers AG, *not available commercially*). For each patient, 2 distinct reconstructions with a temporal resolution of 125 ms and 66 ms were generated using a 512 × 512 matrix and a 200 × 200 mm^2^ field of view.^7^ Reconstruction parameters were uniform across reconstructions: UHR mode with 0.2 mm slice thickness and increment, Bv64 kernel, and quantum iterative reconstruction level 4.

To enable reconstructions at different temporal resolutions, images were first generated using all available data from both measurement systems in dual-source mode. Due to the 95-degree angular offset between the 2 x-ray tubes, slightly more than a quarter rotation of data in parallel geometry (following rebinning from fan-beam geometry) sufficed to generate 180 degrees of projection data, yielding a temporal resolution of 66 ms at the isocenter for a gantry rotation time of 0.25 seconds. In a second step, to simulate single-source image acquisition, only data from 1 measurement system were used. In this configuration, half a rotation in parallel geometry was required, resulting in a temporal resolution of 125 ms at the same gantry rotation speed.

Both prospective ECG-triggered and retrospective ECG-gated acquisitions were included. For both modes, the 66 ms and 125 ms reconstructions were generated from the same raw projection data using identical reconstruction parameters and the same motion-minimized cardiac phase, ensuring that temporal resolution was the only variable between reconstructions. In all cases, the cardiac phase with the least motion artifacts—typically mid-diastole at ~70% of the R-R interval, or end-systole at ~40% if necessary—was selected by an experienced cardiovascular radiologist [(V.M.), with years of experience in cardiovascular CT]. An additional ±10% reconstruction padding around the selected phase was applied to ensure complete data coverage for both reconstructions, maintaining full image quality and identical dose conditions.^9^

### Deep Learning–based Algorithm for Automated Coronary Plaque Quantification

This study employed a novel DL-based prototype software (Coronary Analysis Prototype, version 1.0.6, Siemens Healthineers AG) built upon previous versions.^13–15^ The system provides DL-based, fully automated quantification of coronary plaques from single-phase CCTA reconstructions, focusing on calcified, noncalcified high-density (HD), and noncalcified low-density (LD) plaque components. The software operates in several steps. First, patient-specific coronary centerline trees are automatically traced from CCTA images using an approach previously described.^16^ In the second step, the coronary centerlines are labeled according to an 18-segment model proposed by the Society of Cardiovascular Computed Tomography (SCCT) using a 3D image-to-image DL model.^13^ In the third step, a multitask deep learning approach is applied to perform automated lesion detection and segmentation of the coronary lumen and vessel wall.^13^ Vessel wall segmentation was trained using an approach analogous to the lumen model in Andre et al,^13^ based on an independent data set of 1337 CCTA cases acquired with EID-CT systems. In the final step, plaque volume is quantified within the region between the segmented lumen and vessel wall using predefined Hounsfield Unit (HU) thresholds: noncalcified LD plaque (−30 to 30 HU), noncalcified HD plaque (30 to 350 HU), and calcified plaque (350 and above) at 120 kVp.^15,17^ Coronary segments with a vessel diameter below 1 mm are excluded from plaque quantification.

After automatic processing is completed, results are displayed as color-coded overlays on the coronary centerlines and curved planar reformations, with plaque components visually differentiated. Short-axis images along the lesion are shown at the proximal origin, point of maximal stenosis, and distal end, with vessel wall and lumen contours overlaid in white and orange, respectively, to illustrate plaque and lumen boundaries. A graphical plot of mean vessel diameter along the vessel length complements these visualizations. Stenosis metrics, including minimum lumen diameter and diameter stenosis grade, are provided. Editing tools allow users to adjust all segmentations and measurements as needed.

### Image Analyses With the Deep Learning–based Software

In all included patients, plaque analysis was restricted to coronary segments with adequate image quality in the selected cardiac phase. No segments were excluded following visual inspection, and all analyzed lesions were located in motion-free regions suitable for automated quantification. The deep learning–based coronary plaque analysis algorithm was applied to both the 66 ms (high temporal resolution) and 125 ms (low temporal resolution) reconstructions for each patient by a cardiovascular radiologist with 5 years of experience in cardiovascular CT (K.K.). To evaluate reproducibility, the algorithm was rerun 3 times in 10 randomly selected patients by a cardiovascular imaging expert with 20 years of experience in cardiovascular CT (H.A.), who also independently reviewed all segmentations and plaque classifications for anatomic accuracy and consistency.

#### Evaluation of Entire Coronary Vessels

The automated segmentations of the left anterior descending artery (LAD), left circumflex artery (CX), and right coronary artery (RCA) were used from the ostium of the respective vessel down to a vessel diameter of 1.5 mm, which was chosen as the cutoff point according to the Coronary Artery Disease—Reporting and Data System (CAD-RADS) 2.0.^18^ The left main artery was included in the LAD.

For each vessel, total plaque volume (sum of calcified and noncalcified), calcified plaque volume, noncalcified plaque volume (HD + LD), HD plaque volume, LD plaque volume, and lumen volume were recorded.

#### Lesion-specific Evaluation

The coronary lesion with the greatest longitudinal extent was identified in the LAD, CX, and RCA for each patient in the 66 ms reconstruction. To ensure comparability, lesion segmentation was standardized by aligning both reconstructions to the proximal and distal extent of the lesion as defined in the 66 ms data set. For each matched lesion, total, calcified, and noncalcified plaque volumes (including HD and LD components) were quantified. Lumen volume was measured, and the diameter stenosis grade was calculated as the minimum lumen diameter within the lesion divided by the proximal reference diameter, expressed as a percentage.

### Statistical Analyses

Demographic and clinical data were summarized using descriptive statistics. Normality was assessed for each variable using the Shapiro-Wilk test. Discrete variables were reported as counts (percentages). Continuous variables were reported as mean ± SD or median (interquartile ranges), as appropriate. As plaque volume, lumen volume, and stenosis grade were not normally distributed, comparisons between reconstructions were performed using paired Wilcoxon signed-rank tests. The Benjamini-Hochberg procedure was applied to adjust for multiple comparisons. Visualizations were presented as box-and-whisker plots, which were chosen to display the distribution and direction of paired differences across the patient cohort and to illustrate systematic differences between reconstructions. A 2-sided *P* value < 0.05 was considered statistically significant. All analyses were performed using R version 4.4.0 (R Core Team, Vienna, Austria).

## RESULTS

### Study Population

**FIGURE 1:**
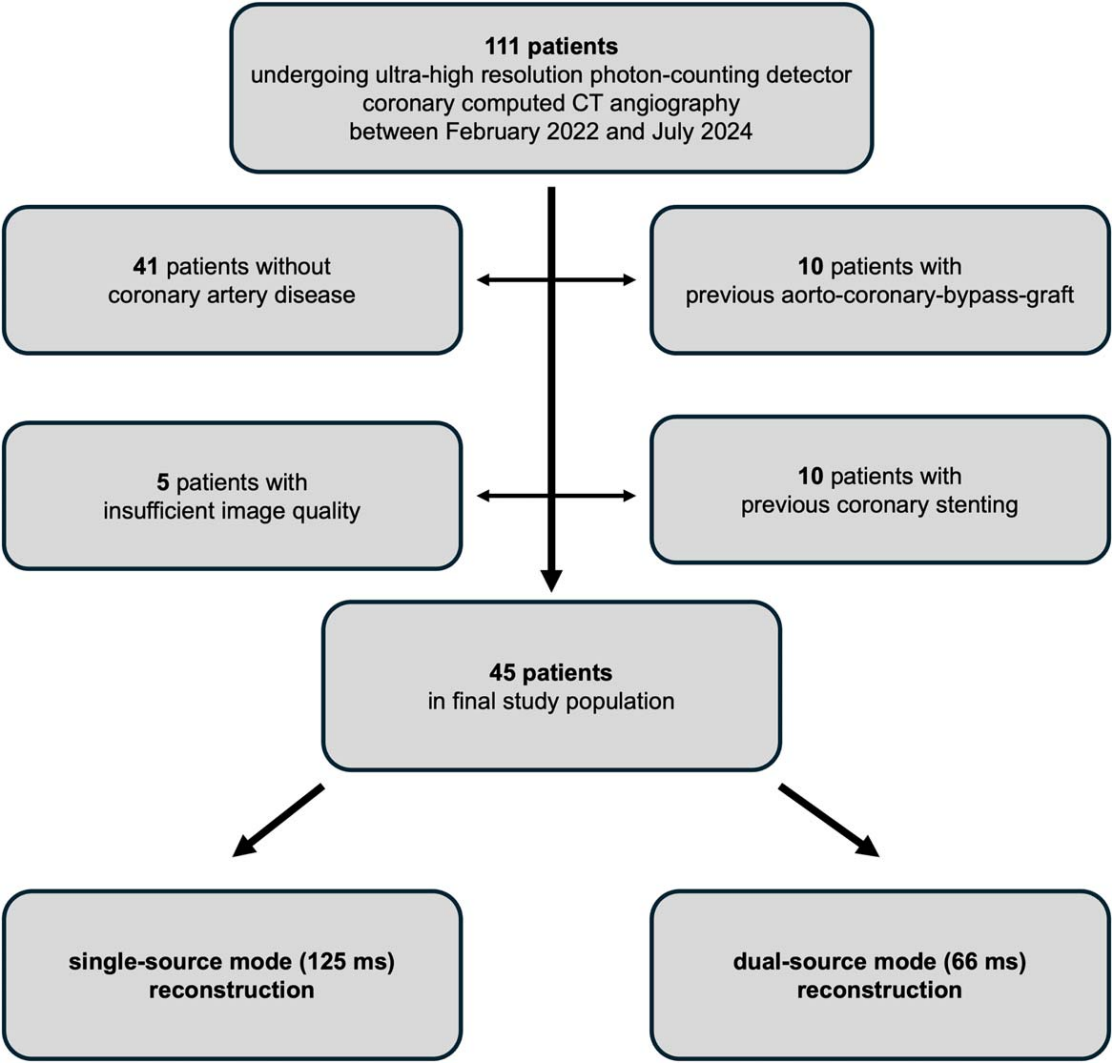
Flowchart of the study.

A total of 111 patients undergoing UHR CCTA with PCD-CT between February 2022 and July 2024 were screened for study inclusion. After applying exclusion criteria, 45 patients were included in the final study population. Exclusions were due to the absence of CAD (n = 41), prior revascularization with bypass grafting (n = 10) or coronary stenting (n = 10), and insufficient image quality caused by excessive motion (n = 4) or metallic artifacts (n = 1) (Fig. 1).

The final study cohort included 31 men (68.9%) and 14 women (31.1%) with a mean age of 73 ± 9 years and a mean body mass index of 25.5 ± 4.5 kg/m^2^. The mean heart rate during UHR CCTA was 68 ± 11 beats per minute (bpm). The median coronary artery calcium score was 281 (interquartile range: 104, 541). Coronary arterial dominance was right-dominant in 39 patients (86.7%), left-dominant in 5 patients (11.1%), and codominant in 1 patient (2.2%). (Table 1).

**TABLE 1:** Patient Demographics and Imaging Parameters

Demographics	n=45 Patients
Sex, n (%)	
Male	31 (68.9)
Female	14 (31.1)
Age (y)	73 ± 9
Body mass index (kg/m^2^)	25.5 ± 4.5
Arterial hypertension, n (%)	32 (71.1)
Coronary arterial dominance, n (%)	
Right dominant	39 (86.7)
Left dominant	5 (11.1)
Codominant	1 (2.2)
Dyslipidemia, n (%)	22 (48.9)
Diabetes, n (%)	6 (13.3)
History of smoking, n (%)	17 (37.8)
Chronic obstructive pulmonary disease, n (%)	3 (6.7)
Chronic kidney disease, n (%)	11 (24.4)
Imaging parameters for coronary CT angiography	
Heart rate (bpm)	68 ± 11
Acquisition mode, n (%)	
Sequential	15 (33.3)
Spiral	30 (66.7)
Volume weighted CT dose index (mGy)	36.5 (31.0, 43.5)
Coronary artery calcium score	281 (104, 541)

Values are provided in counts (percentages), mean ± SD or median (interquartile range) as appropriate.

### Vessel-based Plaque Volume Quantification

Of 135 coronary arteries, 119 (88.1%) showed atherosclerotic plaques and were thus included in the analysis: 45/45 LAD (100%), 39/45 RCA (87%), and 35/45 CX (78%). The median segmented vessel length was 112.7 mm (91.0, 141.6) in the LAD, 128.5 mm (98.0, 157.2) in the RCA, and 79.8 mm (61.5, 110.1) in the CX.

DL-based plaque segmentation was feasible in all patients and no manual adjustments were necessary. All segmentations and plaque classifications were visually reviewed and accepted as anatomically correct by the expert reader. Reproducibility analysis yielded identical plaque and lumen measurements across repeated runs (*P* > 0.999).

Plaque volumes of entire coronary vessels were higher in 125 ms reconstructions compared with 66 ms reconstructions in most patients. Total plaque volumes were higher in 125 ms reconstructions in 28 patients (62%), calcified plaque volumes in 32 patients (71%), and noncalcified plaque volumes in 28 patients (62%).

Total plaque volumes were significantly higher in the 125 ms compared with the 66 ms reconstructions in the LAD (125 ms: 271.8 mm^3^ vs. 66 ms: 250.4 mm3, *P* < 0.001), while differences in the CX (125 ms: 159.8 mm^3^ vs. 66 ms: 151.9 mm^3^, *P* = 0.122) and RCA (125 ms: 317.5 mm^3^ vs. 66 ms: 305.6 mm^3^, *P* = 0.124) were not statistically significant. The total plaque volumes of all vessels were significantly higher in the 125 ms reconstructions (125 ms: 681.3 mm^3^ vs. 66 ms: 647.8 mm^3^, *P* < 0.05) (Table 2).

**TABLE 2:** Plaque Volume Quantification of Entire Coronary Vessels in High and Low Temporal Resolution Data Sets

Plaque Type	Reconstruction: 66 ms Plaque Volume (mm^3^)	Reconstruction: 125 ms Plaque Volume (mm^3^)	*P* *
Calcified-LAD	83.9 (33.4, 170.7)	97.3 (39.6, 202.1)	<0.001
Calcified-CX	42.9 (18.6, 82.5)	47.8 (20.1, 94.6)	0.058
Calcified-RCA	71.2 (30.0, 156.4)	75.1 (33.1, 172.6)	0.019
Calcified-combined	222.3 (96.6, 389.3)	241.1 (106.5, 414.8)	0.001
Noncalcified high-density-LAD	148.1 (98.0, 202.5)	160.5 (104.9, 221.5)	0.001
Noncalcified high-density- CX	91.2 (56.1, 142.5)	97.4 (60.9, 148.2)	0.094
Noncalcified high-density-RCA	170.4 (105.4, 268.7)	178.4 (113.3, 273.2)	0.117
Noncalcified high-density-combined	347.0 (227.5, 474.8)	361.3 (235.5, 491.1)	0.025
Noncalcified low-density-LAD	12.2 (9.1, 19.4)	13.5 (9.3, 20.3)	0.024
Noncalcified low-density-CX	7.6 (4.6, 11.9)	8.0 (4.7, 12.3)	0.523
Noncalcified low-density-RCA	16.4 (10.6, 26.5)	15.8 (10.5, 26.0)	0.988
Noncalcified low-density-combined	37.8 (25.5, 50.3)	37.4 (26.0, 50.5)	0.684
Noncalcified total-LAD	159.4 (108.4, 220.9)	172.1 (113.1, 233.1)	0.002
Noncalcified total-CX	100.6 (62.7, 150.3)	107.5 (66.0, 158.7)	0.101
Noncalcified total-RCA	189.2 (118.0, 291.2)	195.4 (121.8, 296.2)	0.147
Noncalcified total-combined	401.6 (267.5, 519.8)	414.2 (278.4, 535.4)	0.041
Total-LAD	250.4 (164.8, 357.0)	271.8 (174.6, 386.6)	<0.001
Total-CX	151.9 (88.4, 234.6)	159.8 (95.3, 247.8)	0.122
Total-RCA	305.6 (186.1, 438.5)	317.5 (195.3, 457.2)	0.124
Total-combined	647.8 (436.5, 899.3)	681.3 (449.5, 921.3)	0.023

Values are provided in median (interquartile range). Plaque volumes are provided for 4 plaque types: calcified, noncalcified high-density, noncalcified low-density; noncalcified total plaque represents the aggregate of high- and low-density plaque, and total represents the aggregate of all plaques. Plaque volumes are further specified for the 3 main coronary arteries segmented from the coronary ostia to 1.5 mm vessel diameter: left anterior descending (LAD), left circumflex (CX), and right coronary artery (RCA); combined represents the aggregate of all coronary vessels.

* *P* value after paired Wilcoxon signed-rank tests and Benjamini-Hochberg correction for multiple testing.

Stratified analysis by plaque composition showed significantly higher calcified plaque volumes in 125ms compared with 66 ms reconstructions (125 ms: 241.1 mm^3^ vs. 66 ms: 222.3 mm3, *P* < 0.001). Noncalcified plaque volumes, defined as the sum of HD and LD components, were also significantly higher (125 ms: 414.2 mm^3^ vs. 66 ms: 401.6 mm^3^, *P* < 0.05). HD plaque volumes were higher (125 ms: 361.3 mm^3^ vs. 66 ms: 347.0 mm^3^, *P* < 0.05), while LD plaque volumes showed no significant difference (125 ms: 37.4 mm^3^ vs. 66 ms: 37.8 mm3, *P* = 0.684) (Table 2).

**FIGURE 2:**
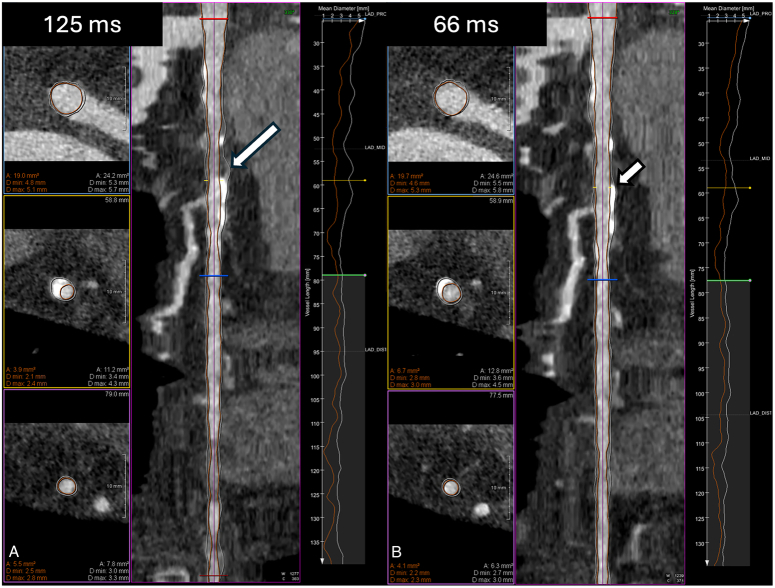
Effect of temporal resolution on deep learning–based coronary plaque quantification. Eighty-three-year-old woman with a coronary calcium score of 488. A, B, display curved planar reformation (CPR) and axial slices of the left anterior descending artery, as well as a graphical representation of mean vessel diameter plotted against the vessel length. Axial slices illustrate the lesion at the proximal origin (top, red), point of maximal stenosis (middle, green marker), and distal end (bottom, blue)—as indicated by corresponding color markers on the CPR. Vessel wall and lumen contours are overlaid in white and orange, respectively. The 125 ms reconstruction (A) shows motion-related blooming of a partially calcified lesion (long arrow), leading to overestimation of vessel wall plaque and underestimation of lumen area, compared with the 66 ms reconstruction (B), where calcifications appear sharper and vessel boundaries are more accurately delineated (short arrow).

**FIGURE 3:**
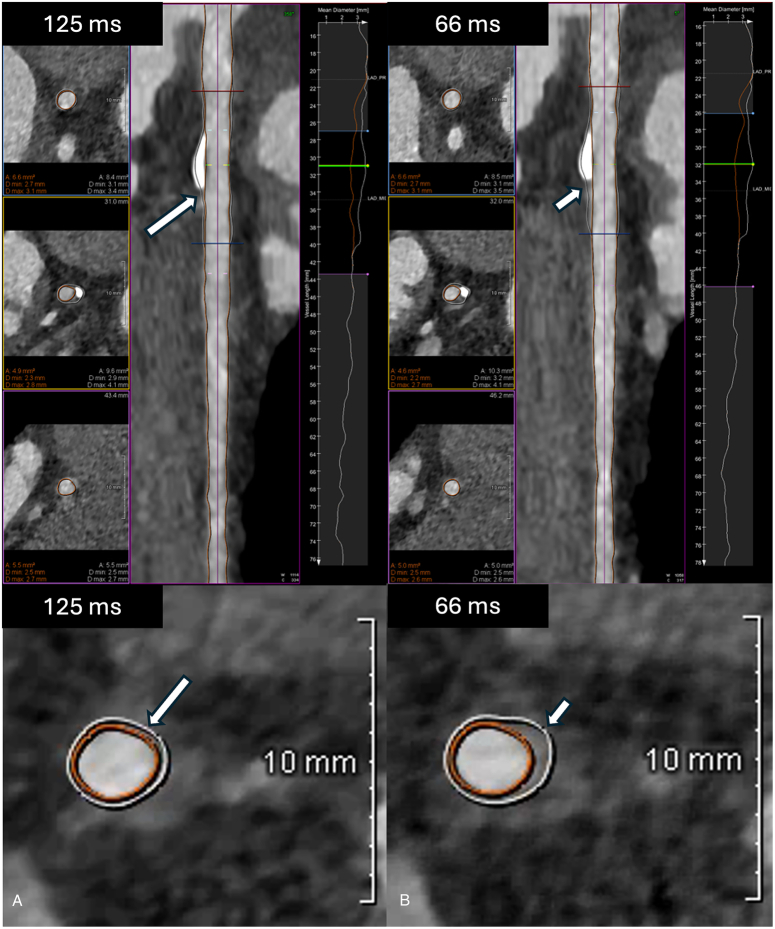
Increased deep learning–based coronary plaque volume delineation in higher temporal resolution data sets. Ninety-three-year-old woman with a coronary calcium score of 40. A, B, display curved planar reformation (CPR) and axial slices of the left anterior descending artery, as well as a graphical representation of mean vessel diameter plotted against the vessel length. Axial slices illustrate the lesion at the proximal origin (top, red), point of maximal stenosis (middle, green marker), and distal end (bottom, blue)—as indicated by corresponding color markers on the CPR. Vessel wall and lumen contours are overlaid in white and orange, respectively. In contrast to the general trend, the 66 ms reconstruction (B) resulted in a slightly larger segmented plaque burden (short arrow) compared with the 125 ms reconstruction (A, long arrow), likely due to improved delineation of noncalcified plaque enabled by higher temporal resolution. The vessel wall and lumen profile curves (white and orange in bottom axial images) are tighter in the 125 ms reconstruction, leading to a smaller segmented plaque volume.

Representative image examples of 2 patients with images reconstructed at different temporal resolutions and analyzed with the DL-based software tool are shown in Figures 2 and 3.

### Lesion-based Plaque Volume Quantification

Lesion-based plaque volumes were higher in 125 ms reconstructions compared with 66 ms reconstructions in most patients. Total plaque volumes were higher in 125 ms reconstructions in 44 patients (98%), calcified plaque volumes in 42 patients (93%), and noncalcified plaque volumes in 42 patients (93%).

Total plaque volumes were significantly higher in 125 ms reconstructions compared with 66 ms reconstructions in the LAD (125 ms: 179.5 mm^3^ vs. 66 ms: 161.0 mm^3^, *P* < 0.001), CX (125 ms: 131.7 mm^3^ vs. 66 ms: 115.0 mm^3^, *P* < 0.01), and RCA (125 ms: 177.1 mm^3^ vs. 66 ms: 161.6 mm^3^, *P* < 0.001). The total plaque volumes of all vessels were significantly higher in the 125 ms reconstructions (125 ms: 447.3 mm^3^ vs. 66 ms: 414.9 mm^3^, *P* < 0.001) (Table 3).

**TABLE 3:** Lesion-specific Coronary Plaque Volume Quantification in High and Low Temporal Resolution Data Sets

Plaque Type	Reconstruction: 66 ms Plaque Volume (mm^3^)	Reconstruction: 125 ms Plaque Volume (mm^3^)	*P* *
Calcified-LAD	61.7 (24.6, 126.7)	67.0 (31.6, 132.9)	<0.001
Calcified-CX	34.7 (13.0, 82.6)	38.1 (15.3, 86.6)	0.034
Calcified-RCA	37.6 (11.3, 92.4)	43.5 (11.7, 95.0)	<0.001
Calcified-combined	102.7 (51.4, 240.0)	105.2 (66.7, 254.3)	<0.001
Noncalcified high-density LAD	86.3 (54.3, 148.5)	97.9 (56.5, 166.1)	<0.001
Noncalcified high-density-CX	69.5 (39.3, 106.0)	70.2 (45.5, 117.3)	0.001
Noncalcified high-density RCA	98.4 (60.7, 141.3)	114.0 (82.6, 192.0)	<0.001
Noncalcified high-density-combined	220.5 (115.5, 359.4)	286.3 (164.6, 403.7)	<0.001
Noncalcified low-density-LAD	8.3 (5.1, 16.4)	8.4 (5.0, 14.9)	0.002
Noncalcified low-density-CX	5.0 (3.7, 10.0)	5.3 (3.5, 10.7)	0.281
Noncalcified low-density-RCA	10.2 (5.4, 15.3)	10.9 (8.1, 16.7)	0.001
Noncalcified low-density-combined	22.5 (12.5, 35.4)	24.6 (14.7, 36.9)	<0.001
Noncalcified total-LAD	94.7 (60.6, 162.8)	109.0 (61.6, 184.2)	<0.001
Noncalcified total-CX	74.8 (43.4, 115.3)	75.5 (49.3, 129.3)	0.001
Noncalcified total-RCA	109.4 (66.5, 156.1)	124.8 (90.3, 211.0)	<0.001
Noncalcified total-combined	237.6 (130.2, 394.8)	309.3 (178.2, 436.5)	<0.001
Total-LAD	161.0 (87.9, 309.5)	179.5 (103.3, 333.4)	<0.001
Total-CX	115.0 (62.9, 168.1)	131.7 (70.0, 182.7)	0.002
Total-RCA	161.6 (87.2, 239.8)	177.1 (104.6, 330.9)	<0.001
Total-combined	414.9 (160.2, 668.2)	447.3 (233.3, 712.5)	<0.001

Values are provided in median (interquartile range). Plaque volumes are provided for 4 plaque types: calcified, noncalcified high-density, noncalcified low-density; noncalcified total plaque represents the aggregate of high- and low-density plaque, and total represents the aggregate of all plaques. Plaque volumes are further specified for the largest lesion in the 3 main coronary arteries: left anterior descending (LAD), left circumflex (CX), and right coronary artery (RCA); combined represents the aggregate of LAD, CX, and RCA.

* *P* value after paired Wilcoxon signed-rank tests and Benjamini-Hochberg correction for multiple testing.

**FIGURE 4:**
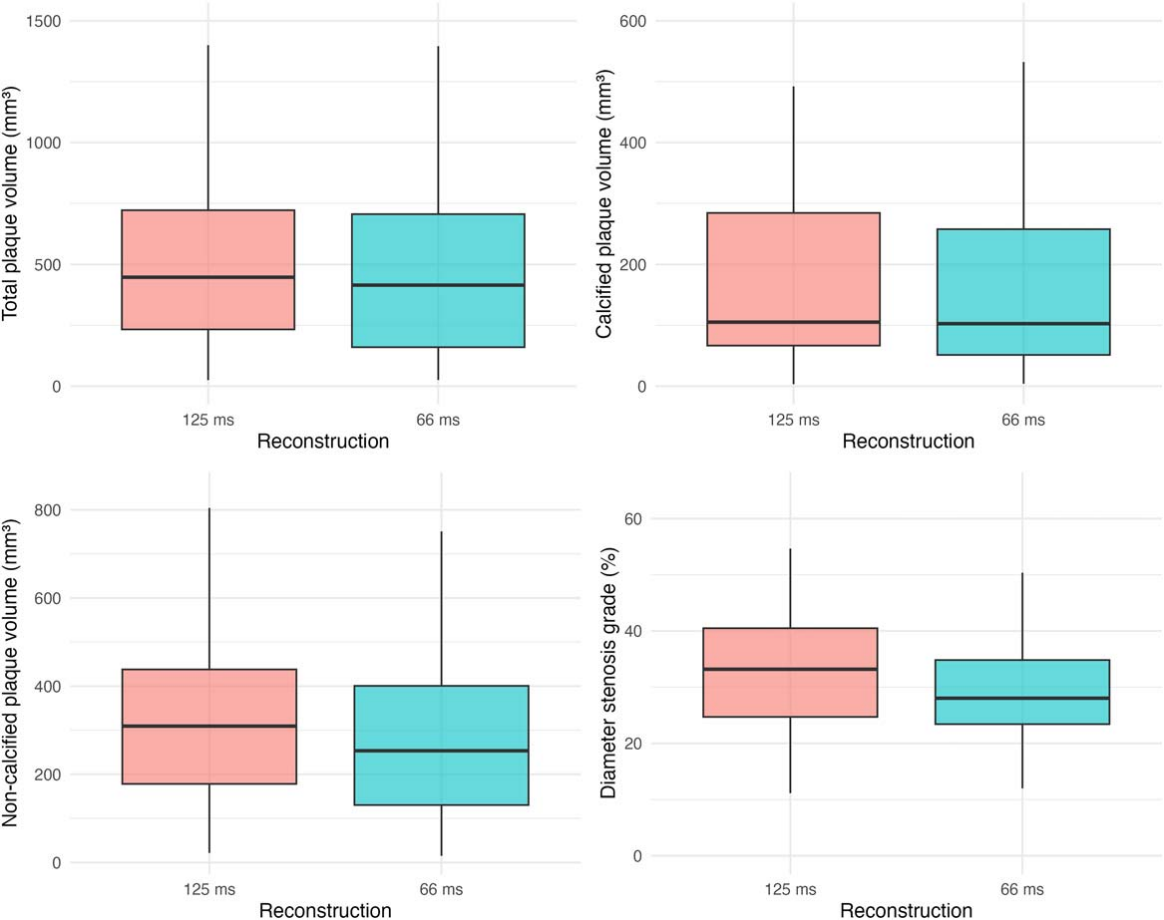
Boxplots for lesion-specific deep learning–based coronary plaque volume and diameter stenosis grade quantification in different temporal resolution data sets. Boxplots compare lesion-specific measurements between single-source (125 ms) and dual-source (66 ms) reconstructions, including total plaque volume, calcified plaque volume, noncalcified plaque volume, and diameter stenosis grade. Each boxplot shows the median, interquartile range, and overall distribution.

Stratified analysis by plaque composition showed significantly higher calcified plaque volumes in 125 ms reconstructions compared with 66 ms reconstructions (125 ms: 105.2 mm^3^ vs. 66 ms: 102.7 mm^3^, *P* < 0.001). Noncalcified plaque volumes, defined as the sum of HD and LD components, were also significantly higher (125 ms: 309.3 mm^3^ vs. 66 ms: 237.6 mm^3^, *P* < 0.001). Both HD (125 ms: 286.3 mm^3^ vs. 66 ms: 220.5 mm^3^, *P* < 0.001) and LD plaque volumes (125 ms: 24.6 mm^3^ vs. 66 ms: 22.5 mm^3^, *P* < 0.001) were significantly higher in the 125 ms reconstructions (Table 3, Figure 4).

### Coronary Lumen Volume and Diameter Stenosis Quantification

Lumen volume quantification of entire coronary vessels revealed significantly lower volumes in the 125- ms compared with the 66 ms reconstructions in the LAD (801.8 mm^3^ vs. 819.4 mm^3^, *P* < 0.001), CX (522.9 mm^3^ vs. 541.1 mm^3^, *P* < 0.01), and RCA (856.4 mm^3^ vs. 887.0 mm^3^, *P* < 0.01) (Supplemental Table, Supplemental Digital Content 1, http://links.lww.com/RLI/B57).

The coronary lumen volumes at the level of the lesions were significantly lower for the 125 ms reconstructions in the LAD (289.8 mm^3^ vs. 298.9 mm^3^, *P* < 0.01), CX (218.9 mm^3^ vs. 225.5 mm^3^, *P* = 0.002), and RCA (298.5 mm^3^ vs. 329.4 mm^3^, *P* < 0.001) (Supplemental Table, Supplemental Digital Content 1, http://links.lww.com/RLI/B57).

Median diameter stenosis grades were higher in the 125 ms reconstructions in 33 patients (73.3%) (Figure 4). Median diameter stenosis grades were significantly higher in the average of all vessels (125 ms: 35.4% vs. 66 ms: 28.1%, *P* < 0.01) and in the LAD alone (125 ms: 36.2% vs. 66 ms: 35.5%, *P* < 0.05), but not in the CX (*P* = 0.136) or RCA alone (*P* = 0.137) (Supplemental Table, Supplemental Digital Content 1, http://links.lww.com/RLI/B57).

## DISCUSSION

In this study, we evaluated a novel, on-site, fully automated DL-based software for coronary plaque quantification. The algorithm was applied to UHR CCTA data sets acquired with a dual-source PCD-CT and reconstructed at two different temporal resolutions (66 ms vs. 125 ms) for each patient. These effects were most pronounced at the plaque level. The algorithm demonstrated excellent reproducibility, yielding identical results across repeated runs in a subset of patients, and all outputs were confirmed as anatomically consistent by expert review. Reconstructions at lower temporal resolution (125 ms) systematically overestimated the total, calcified, and noncalcified plaque volumes as well as stenosis severity. Our findings highlight that temporal resolution substantially impacts DL-derived quantitative metrics in UHR CCTA and emphasize the importance of resolution-aware algorithm development and protocol standardization.

While numerous DL-based solutions for plaque quantification exist, most were developed using EID-CT data and have not been validated for use in UHR CCTA. Off-site, cloud-based platforms such as Cleerly (Cleerly Labs) and HeartFlow (HeartFlow Inc.) rely on convolutional neural networks trained on large data sets from EID-CT and have been used in multicenter outcome studies.^5,12,19,20^ AutoPlaque (Autoplaque 3.0, Cedars-Sinai Medical Center) is a semi-automated, on-site, supervised-learning system requiring user interaction.^2,4^

The system used in our study represents the latest generation of DL-based coronary analysis tools, being a successor of earlier prototypes previously evaluated by André et al^13^ and Weichsel et al.^21^ While these previous versions demonstrated the feasibility of on-site integration and partially automated workflows, they still required operator interaction for final adjustments. The current version advances this concept by providing fully automated case preparation and end-to-end quantification of total plaque burden, plaque subcomponents (calcified, HD, and LD noncalcified), and stenosis grading, with editing tools available if needed. The results were technically robust, reproducible across repeated runs, required no manual corrections, and consistently matched the anatomic coronary morphology confirmed by expert review. Such a fully automated system could be highly valuable in clinical practice, as total plaque volume is a well-established predictor of adverse cardiovascular events.^22^

We used UHR CCTA data acquired with a dual-source PCD-CT, offering 0.2 mm resolution, which has been indicated to refine coronary plaque characterization.^23^ The UHR CCTA modes improve vessel sharpness and reduce blooming artifacts compared with EID-CT, particularly benefiting the assessment of calcified plaques.^7^ Vecsey-Nagy et al^24^ showed that UHR CCTA yields lower plaque volumes and higher reproducibility than EID-CT in a cohort similar to ours (patients before TAVR), although their analysis required extensive manual segmentation and correction.

Temporal resolution is a critical parameter for motion-sensitive CCTA, an issue that is pronounced at higher spatial resolution.^9^ This also holds true for coronary calcium scoring, where motion-related vessel wall blurring in images with lower temporal resolution leads to overestimation of calcified plaque burden.^25^ Our study confirms and extends these prior observations by providing quantitative evidence that higher temporal resolution improves plaque characterization not only in conventional assessments but also in fully automated DL-based plaque and stenosis quantification.

This study has several limitations. First, it was conducted at a single center on a single PCD-CT scanner, which may limit generalizability to other institutions, scanner models, and reconstruction platforms. Second, plaque and stenosis measurements were not validated against a reference standard such as intravascular ultrasound or optical coherence tomography. Third, the DL-based quantification tool was a research prototype not yet cleared for clinical use, and the processing time was not systematically assessed, as it depends on local hardware, and this prototype was installed on a dedicated research platform. Fourth, this study was specifically designed to isolate the effect of temporal resolution and therefore did not evaluate the influence of other technical parameters such as reconstruction kernels, noise levels, or postprocessing algorithms on plaque quantification.^26^ Fifth, the DL-based software was developed by the same vendor as the PCD-CT system and has so far only been applied to data sets from this platform. Finally, the study population consisted of patients undergoing transcatheter aortic valve replacement planning, a cohort with high calcium burden and predominantly calcified plaques. Together with the modest sample size of 45 patients, this may limit generalizability, and results may differ in other populations—such as those with acute chest pain—where mixed or noncalcified plaques are more common.^27^ Larger, multicenter studies in diverse cohorts are needed to confirm and extend these findings.

In conclusion, this study evaluated a novel DL-based tool with automatic case preparation for quantitative coronary plaque and stenosis assessment in UHR CCTA data sets. The algorithm was technically robust and reproducible, delivering anatomically consistent outputs without manual correction. Reconstructions with lower temporal resolution (125 ms) systematically overestimated plaque burden compared with higher temporal resolution (66 ms), underscoring that protocol standardization is essential for reliable DL-based plaque quantification and stenosis grading.

## Supplementary Material
