# Supplementary material for: Deep Learning–based Automated Coronary Plaque Quantification: First Demonstration With Ultra-high Resolution Photon-counting Detector CT at Different Temporal Resolutions
Source: Invest Radiol. 2025 Aug 22;61(5):341–9. doi: 10.1097/RLI.0000000000001233 (PMC13045810; doi:10.1097/RLI.0000000000001233)
Supplement: Supplementary file 1 [file rli-61-341-s001.docx]

***Supplemental Table.*** Coronary lumen volume and diameter stenosis grade quantification

| **Plaque type** | **Reconstruction: 66 ms**  Lumen volume (mm^3^)  Diameter stenosis grade (%) | **Reconstruction: 125 ms**  Lumen volume (mm^3^)  Diameter stenosis grade (%) | | **P-value*** | |
| --- | --- | --- | --- | --- | --- |
| Lumen volume – LAD^1^ | 819.4 (588.5, 1143.2) | 801.8 (573.5, 1094.2) | <0.001 | |  |
| Lumen volume – CX^1^ | 541.1 (395.2, 707.3) | 522.9 (383.6, 684.1) | 0.001 | |  |
| Lumen volume – RCA^1^ | 887.0 (640.4, 1132.6) | 856.4 (609.3, 1091.7) | 0.001 | |  |
| Lumen volume – combined^1^ | 2233.0 (1673.0, 2853.3) | 2166.3 (1601.6, 2783.1) | <0.001 | |  |
| Lumen volume – LAD^2^ | 298.9 (204.2, 442.1) | 289.8 (177.4, 427.9) | 0.001 | |  |
| Lumen volume – CX^2^ | 225.5 (139.4, 502.0) | 218.9 (119.3, 500.0) | 0.002 | |  |
| Lumen volume – RCA^2^ | 329.4 (124.1, 640.3) | 298.5 (109.7, 617.1) | <0.001 | |  |
| Lumen volume – combined^2^ | 1068.3 (546.8, 1506.8) | 1003.6 (547.0, 1453.1) | <0.001 | |  |
| Diameter stenosis grade – LAD^2^ | 35.5 (25.2, 45.5) | 36.2 (29.7, 51.4) | 0.022 | |  |
| Diameter stenosis grade – CX^2^ | 26.8 (19.2, 43.0) | 27.6 (19.9, 33.6) | 0.136 | |  |
| Diameter stenosis grade – RCA^2^ | 25.9 (17.8, 35.6) | 33.0 (18.9, 39.2) | 0.137 | |  |
| Diameter stenosis grade – combined^2^ | 28.1 (23.7, 34.8) | 35.4 (24.7, 40.5) | 0.003 | |  |

Note.- Values are provided in median (interquartile range). Reconstructions are provided with temporal resolution (ms).

^1^Lumen volumes are specified for the three main coronary arteries segmented from the coronary ostia to 1.5 mm vessel diameter: left anterior descending (LAD), left circumflex (CX) and right coronary artery (RCA); combined represents the aggregate of all vessels.

^2^Lumen volumes and diameter stenosis grades for the largest lesion in the three main coronary arteries: left anterior descending (LAD), left circumflex (CX) and right coronary artery (RCA); combined represents the aggregate (lumen volume) and average (diameter stenosis grade) of LAD, CX and RCA. *P-value after paired Wilcoxon signed-rank tests and Benjamini-Hochberg correction for multiple testing.
